# Immunodominant epitope-specific Th1 but not Th17 responses mediate protection against *Helicobacter pylori* infection following UreB vaccination of BALB/c mice

**DOI:** 10.1038/srep14793

**Published:** 2015-10-05

**Authors:** Bin Li, Li Chen, Heqiang Sun, Wuchen Yang, Jian Hu, Yafei He, Shanshan Wei, Zhuo Zhao, Jinyong Zhang, Haibo Li, Quanming Zou, Chao Wu

**Affiliations:** 1 National Engineering Research Center of Immunological Products, Department of Microbiology and Biochemical Pharmacy, College of Pharmacy, Third Military Medical University, Chongqing 400038, P.R. China; 2 Department of Blood Transfusion, Xinqiao Hospital, Third Military Medical University, Chongqing 400037, P.R. China; 3 Department of Gastroenterology, Xinqiao Hospital, Third Military Medical University, Chongqing 400037, P.R. China

## Abstract

*Helicobacter pylori* (*H. pylori*) infects more than half of the world’s population, causing chronic gastritis, peptic ulcers and gastric cancer. Urease B subunit (UreB), a conserved protein of *H. pylori*, is capable of inducing specific CD4^+^ T-cell responses and provides protection against this infection. Previous studies have confirmed the effectiveness of rUreB subunit vaccines in generating CD4^+^ T-cell-mediated protection, but less is known regarding the roles of different subtypes of T-cell immunity, such as Th1, Th2 and Th17, particularly the immunodominant epitopes inducing specific CD4^+^ T-cell responses, in vaccine-mediated protection. In this study, we demonstrated that the vaccination of BALB/c mice with rUreB resulted in significant antigen-specific Th1 and Th17 immune responses. Importantly, two novel Th epitopes, UreB_317–329_ and UreB_409–421_, which are recognized by a major population of CD4^+^ T cells, were identified in immunized mice. Our results demonstrated that two novel epitopes can simultaneously induce Th1 and Th17 immune responses; however, only the epitope vaccine-induced CD4^+^ T-cells secreting IFN-γ mediated the protection against *H. pylori*; cells secreting IL-17A did not. Taken together, our results suggest that two novel immunodominant epitopes can induce Th1 and Th17 immune responses, but only the induced Th1 lymphocytes mediate protection against *H. pylori*.

*Helicobacter pylori*, a spiral-shaped, Gram-negative bacterium, resides in the gastric mucosa and infects more than half of the world’s population^1^,^2^. Persistent *H. pylori* infections are linked with chronic gastritis, peptic ulcers, gastric mucosa-associated lymphoid tissue lymphoma (MALT) and gastric cancer^3^. However, the protective mechanisms against *H. pylori* infection remain unclear. Many vaccines have been developed to eradicate *H. pylori* by activating the humoral immune response, but its protective effect remains unsatisfactory^4^. Conversely, CD4^+^ T-cell responses contribute to the protection against extracellular bacterial infection^5^,^6^. Thus, a focus on specific CD4^+^ T-cell responses is necessary to recognize the protective mechanism against *H. pylori* infection.

Urease is a main virulence factor associated with the colonization, infection and survival of *H. pylori* in the stomach^7^. Many studies have confirmed that vaccination with Urease B subunit (UreB) provides effective protection against *H. pylori* in mice, monkeys and even humans^8^,^9^. UreB is an excellent vaccine candidate that induces significant CD4^+^ T-cell responses, including Th1/Th2/Th17 immune responses^10^. However, the immunologic mechanisms underlying different types of specific CD4^+^ T-cell responses to UreB have not yet been fully elucidated. The characterization of CD4^+^ T-cell epitopes in UreB and its correlation with protection are vital to understanding anti-*H. pylori* immune responses. In our previous study, three CD4^+^ T-cell epitopes of UreB were identified using a bioinformatics-based prediction approach^11^. However, a systematic identification of the immunodominant epitope-specific CD4^+^ T-cell responses has not yet been attempted.

In this study, we fully characterized the immunodominant CD4^+^ T-cell epitopes of UreB and investigated its protective mechanisms in mice. Our findings demonstrated that UreB significantly increased antigen-specific CD4^+^ T-cell responses and provided protection against *H. pylori* infection. Importantly, two novel Th epitopes, UreB_317–329_ and UreB_409–421_, elicited immunodominant CD4^+^ T cells to secrete IFN-γ and IL-17A. Our results also showed that the two Th epitopes could induce specific Th1 and Th17 responses simultaneously; however, only epitope-specific CD4^+^ T-cells secreting IFN-γ, but not IL-17A, mediated protective immunity against *H. pylori*. Taken together, our findings clarified the role of protective immunity against *H. pylori* infection derived from two immunodominant epitope-specific Th1 responses, but not from Th17 responses, following the subcutaneous vaccination of UreB in mice. These results may provide useful insights into the mechanisms underlying the anti-*H. pylori* immune response.

## Results

### UreB vaccination results in specific Th1 and Th17 immune responses

**Figure 1:**
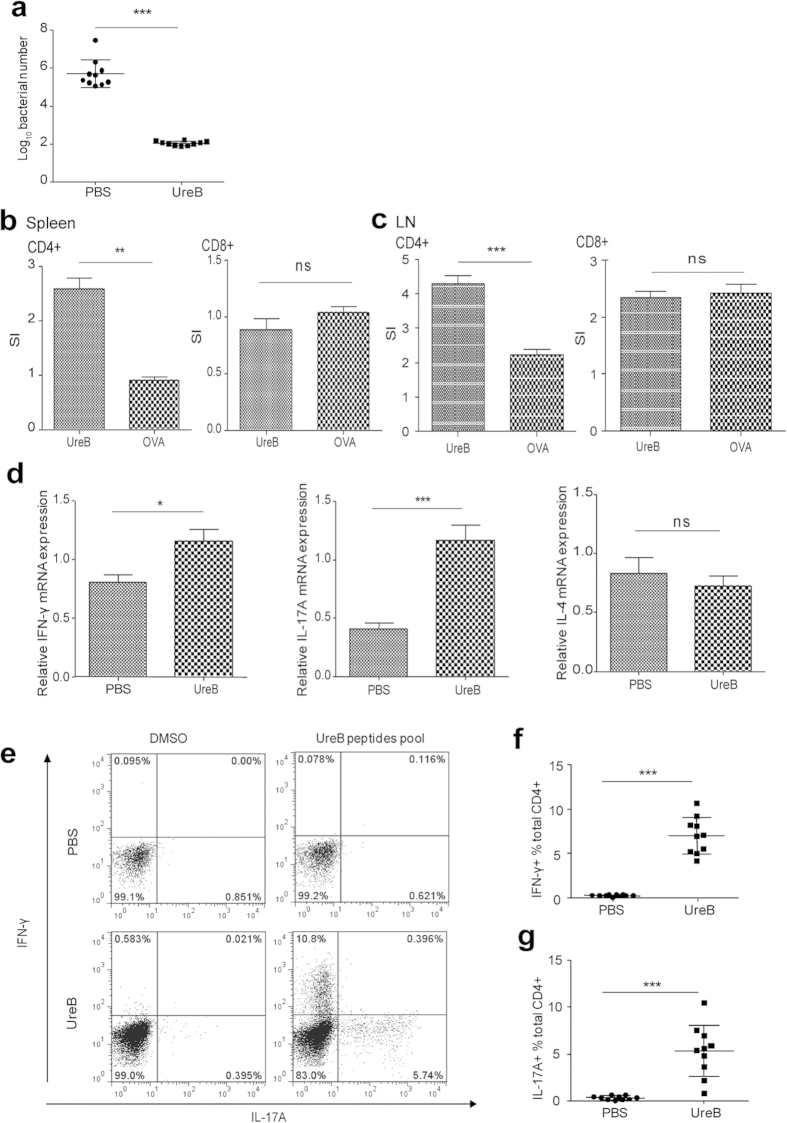
Types of CD4+ T-cell responses elicited via UreB immunization. (**a**) *H. pylori* colonization of the gastric mucosa of UreB-immunized mice at 4 weeks after the last infection. (**b**) Proliferation of CD4^+^ and CD8+ T cells isolated from the spleens of UreB-immunized mice was assessed using the ^3^H-TdR incorporation assay. (**c**) The proliferation of CD4^+^ and CD8+ T cells isolated from stomach-draining lymph nodes of UreB-immunized mice was assessed using the ^3^H-TdR incorporation assay. (**d**) IFN-γ, IL-17A and IL-4 mRNA levels in CD4^+^ T cells isolated from UreB-immunized mice were analyzed. (**e**) Splenic lymphocytes from UreB-immunized mice or PBS controls were cultured *in vitro* in the presence of recombinant UreB. IFN-γ-producing and IL-17A-producing CD4^+^ T cells were assessed using the UreB peptide pool on day 7. (**f**) IFN-γ-producing CD4^+^ T-cell responses of all immunized mice were assayed. (**g**) IL-17A-producing CD4^+^ T-cell responses of all immunized mice were assayed. All results were repeated more than three times. The data are expressed as the mean ± S.D (n = 10). *P < *0.05*, **P < *0.01*, ***P < *0.001*.

To confirm its protective effect against *H. pylori*, BALB/c mice were vaccinated with rUreB emulsified in Freund’s adjuvant and challenged with *H. pylori*. The colonization of *H. pylori* was detected using real-time PCR. Our results demonstrated that gastric *H. pylori* in the rUreB vaccination group significantly decreased compared to PBS controls (Fig. 1a, P < 0.001). To determine which subtype of T lymphocytes take part in the protection against *H. pylori* in UreB immunized mice, CD4^+^ and CD8^+^ T-cells were sorted from spleens of UreB-immunized mice and cocultured with antigen presenting cells (APCs), which were pulsed with the UreB antigen. A ^3^H-TdR incorporation assay was then used to assess cell proliferation. CD4^+^ proliferated in UreB-immunized mice, whereas CD8^+^ T lymphocytes did not (Fig. 1b). Our 3H-TdR proliferation results for lymphocytes in stomach-draining lymph nodes of UreB-immunized mice further supported this conclusion (Fig. 1c). These results indicated that CD4^+^ T cells may play a vital role in protective immunity against *H. pylori* infection.

To determine the subset of CD4^+^ T cells induced by UreB, the cytokine profile of CD4+ lymphocytes isolated from the spleens of UreB-immunized mice was detected using real-time RT-PCR. As shown in Fig. 1d, the mRNA expression level of IFN-γ in immunized mice was significantly higher compared to PBS controls (P < 0.05). Similar differences between the immunized mice and PBS controls were observed for the mRNA expression of IL-17A (P < 0.001) but not IL-4 (Fig. 1d). To further investigate antigen-specific CD4^+^ T-cell responses, specific T lymphocytes of immunized mice were expanded and detected by FCM. The frequency of UreB-specific Th1 and Th17 lymphocytes demonstrated an increase in immunized mice after bulk culture but no significant change in PBS controls. Additionally, few CD4^+^ T cells secreting IFN-γ and IL-17A simultaneously were detected (Fig. 1e). Statistical analyses revealed that the frequencies of antigen-specific Th1 and Th17 lymphocytes were significantly higher in UreB-immunized mice compared to PBS controls (Fig. 1f,g, P < 0.001). These results indicated that UreB vaccination induced strong specific Th1 and Th17 responses, which may protect mice against *H. pylori* infection.

### Two novel CD4^+^ T-cell epitopes of UreB-induced immunodominant Th1 and Th17 responses

**Figure 2:**
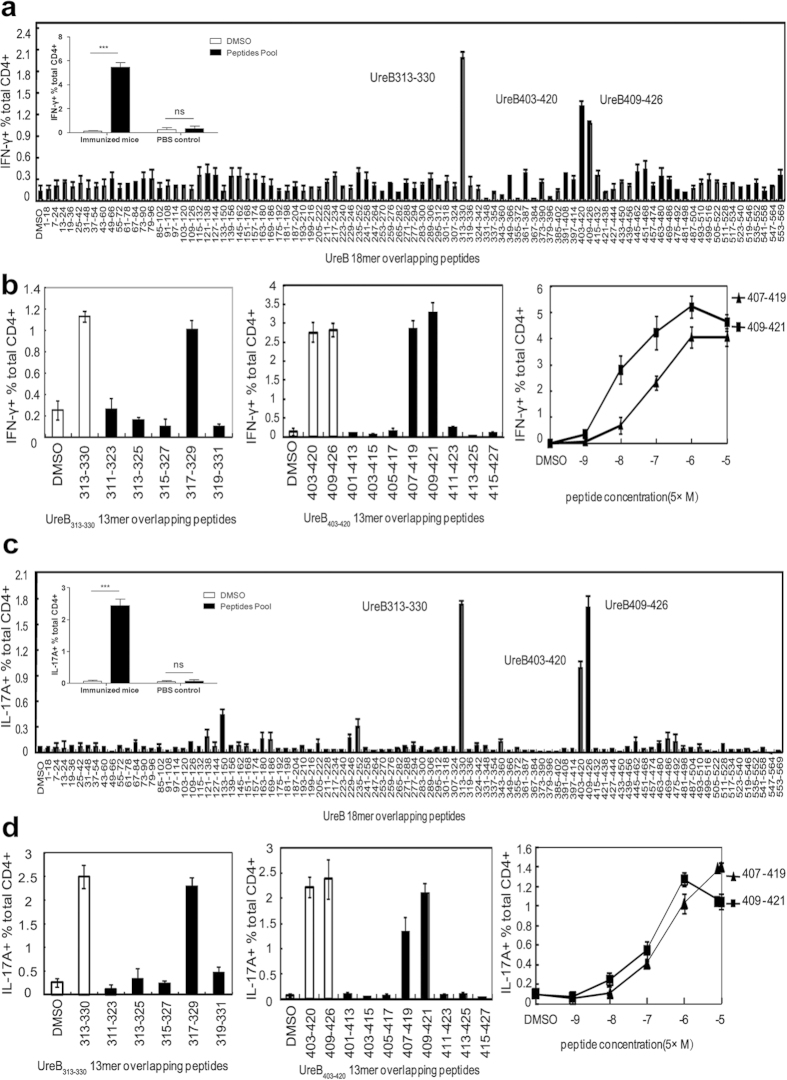
Mapping of UreB immunodominant epitopes. (**a**) UreB-specific T cells expanded from immunized mice were screened for their specific IFN-γ response to 93 overlapping 18-mer UreB peptides at a final concentration of 5 μmol/l in an ICS assay. The locations of the identified 18-mer sequences are shown. The embedding data showed the responses of immunized mice and PBS control mice to the UreB peptide pool. (**b**) The 13-mer overlapping peptides within the UreB_313–330_ 18-mers were screened *(left)*. The 13-mer peptides within UreB_403–420_ and UreB_409–426_ were screened *(middle)*. Peptides were titrated to compare their activities *(right)*. (**c**) Th17 epitopes within the 93 overlapping 18-mer UreB peptides were identified using UreB-specific T cells expanded from immunized mice. The embedding data showed the responses of immunized mice and PBS control mice to the UreB peptide pool. (**d**) 13-mer peptides within UreB_313–330_ 18-mers were screened as described in *b (left)*. The 13-mer peptides within UreB_403–420_ and UreB_409–426_ were screened (middle). Peptides were titrated under serum-free conditions to compare their activities *(right)*. The results are representative of five independent experiments. The data are expressed as the mean ± S.D. (n = 10). ***P < *0.001*.

To investigate which epitopes in UreB induced immunodominant CD4^+^ T-cell responses, splenic lymphocytes of mice immunized with UreB combined with Freund’s adjuvant were isolated and antigen-specific T cells were expanded *in vitro*. On day 7 of bulk culture, cells were harvested and stimulated with 93 overlapping 18-mer peptides. The frequencies of CD4^+^ T cells secreting IFN-γ or IL-17A were then determined using ICS and FCM. The dominant Th1 and Th17 responses were induced by three 18-mer peptides, UreB_313–330,_ UreB_403–420_ and UreB_409–426_ (Fig. 2a,c). The IFN-γ and IL-17A responses from PBS control mice to a UreB peptide pool containing all of the peptides were analyzed. As shown in Fig. 2a,c, the peptide pool induced significant IFN-γ and IL-17A responses in UreB immunized mice. However, no differences were detected between the peptide pool and DMSO control in PBS control mice. These results indicated that no UreB-specific T cells exit in unimmunized mice and no epitopes of UreB could induce a response in unimmunized mice. To further confirm the shorter sequences in the 18-mer peptides, 18-mer peptide-specific T cells were probed using 13-mer peptides with a 2-aa shift within the corresponding 18-mer peptides. UreB_317–329_ elicited the strongest IFN-γ response among five overlapping peptides (Fig. 2b, left). UreB_407–419_- and UreB_409–421_-specific Th1 responses were stronger than other 13-mer peptides in 18-mer peptides UreB_403–420_ and UreB_409–426_ (Fig. 2b, middle). Titration experiments were performed to further confirm the location of the core sequence. As shown in Fig. 2b (right), UreB_409–421_ induced more robust Th1 responses than UreB_407–419_ at different peptide concentrations, suggesting that UreB_409–421_ represented the main peptide that induced the immunodominant Th1 response.

Similarly, the shorter peptides that induced dominant Th17 responses were also identified. Our results indicated that the 13-mer peptides UreB_317–329_ and UreB_409–421_ are also core peptides that induced immunodominant Th17 responses as well as Th1 responses (Fig. 2d).

### Two immunodominant Th epitopes could be naturally processed and presented by DCs via H-2^d^ (I-A) or H-2^d^ (I-E) molecules

**Figure 3:**
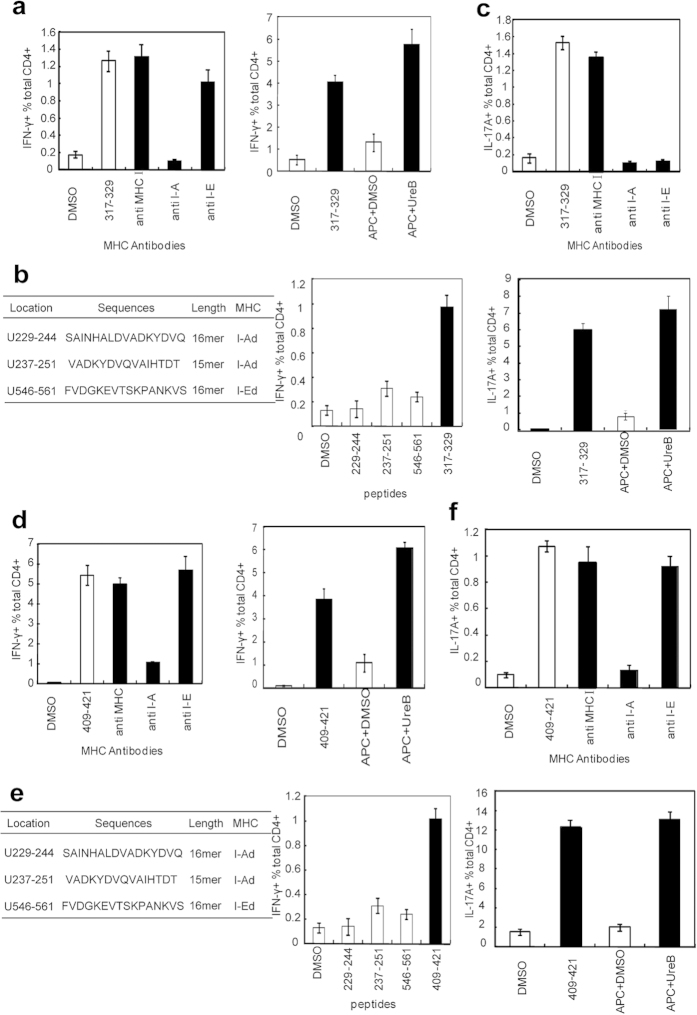
Detailed characterization of immunodominant anti-UreB CD4^+^ T-cell responses. (**a**) MHC antibodies were used to identify the restriction profile of the Th1 epitope UreB_317–329_ *(left)*. Natural processing and presentation of the novel Th1 epitope UreB_317–329_ by DCs *(right)*. (**b**) Information of the three Th1 peptides predicted using a bioinformatics approach, including the location, amino acid sequences, length and MHC restriction profiles confirmed in our previous study *(left)*. IFN-γ-producing CD4^+^ T-cell responses induced by UreB_317–329_ and the predicted peptides were analyzed *(right)*. (**c**) MHC antibodies were used to identify the restriction profile of UreB_317–329_ epitope-specific Th17 responses *(up)*. Natural processing and presentation of the epitope UreB_317–329_ inducing CD4+ T cells secreting IL-17A *(down)*. (**d**) The MHC restriction profile for UreB_409–421_ inducing Th1 responses was determined using MHC antibodies *(left)*. Natural processing and presentation of the epitope UreB_409–421_ inducing CD4+ T cells secreting IFN-γ *(right)*. (**e**) Information of the three Th1 peptides predicted using a bioinformatics approach *(left)*. IFN-γ-producing CD4^+^ T-cell responses induced by UreB_409–421_ and the predicted peptides were analyzed *(right)*. (**f**) The MHC restriction profile for UreB_409–421_ inducing Th17 responses was determined using MHC antibodies *(up)*. The natural processing and presentation of the epitope UreB_409–421_ induced CD4+ T cell secretion of IL-17A *(down)*. The results were repeated five times. The data are expressed as the mean ± S.D (n = 10).

An MHC antibody-blocking assay and natural processing and presentation test were performed to confirm MHC-II molecule subtype binding to the novel Th epitopes and whether these two novel immunodominant Th epitope could be processed and presented by DCs in nature. As shown in Fig. 3a left, the H-2^d^ (I-A) antibody efficiently blocked Th1 responses to UreB_317–329_; the H-2^d^ (I-Ek) and H-2^d^ (I-A) antibodies blocked Th17 responses to UreB_317–329_ (Fig. 3c, up). The responses of UreB_317–329_-specific Th1 and Th17 were strongly activated by DCs pulsed with UreB (Fig. 3a, right, and 3c, down). These results indicated that UreB_317–329_ could be naturally processed and presented by DCs via H-2^d^ (I-A) or H-2^d^ (I-E) to induce specific T-cell responses. Consistent with this finding, we confirmed that another immunodominant epitope, UreB_409–421_, could be naturally processed and presented by DCs to induce specific Th1 and Th17 responses via the H-2^d^ (I-A) molecule (Fig. 3d,f).

We previously predicted and identified three Th peptides, UreB_229–244_, UreB_237–251_ and UreB_546–561_, using a bioinformatics approach^11^. To investigate whether UreB_317–329_ and UreB_409–421_ were more immunodominant epitopes than the three predicted ones, UreB-specific T cells were used to assess the Th1 and Th17 responses to five peptides. As shown in Fig. 3b,e, UreB_317–329_ and UreB_409–421_ elicited stronger IFN-γ and IL-17A responses than the three predicted epitopes.

### Immunodominant Th1 and Th17 responses induced by the same epitope demonstrated different profiles in cytokine secretion and homing receptor expression

**Figure 4:**
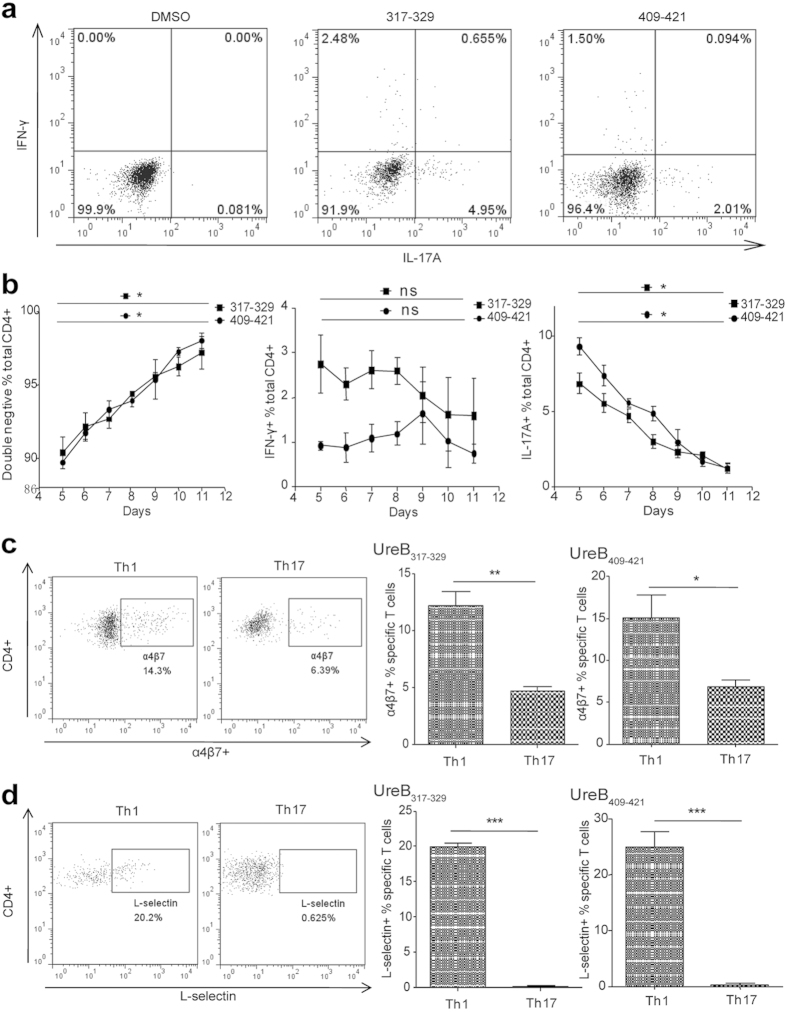
Distinguishing between epitope-specific Th1 and Th17 cells. Splenic lymphocytes of mice immunized with UreB antigen were isolated and cultured in the presence of UreB *in vitro* and then harvested. The responses of the cultured CD4^+^ T cells to peptides UreB_317–329_/UreB_409–421_ and homing receptors expressed by epitope specific T cells were detected via flow cytometry. (**a**) The expanded CD4^+^ T cells secreting IFN-γ and IL-17A were determined simultaneously using the ICS assay after the stimulation of peptides UreB_317–329_ and UreB_409–421_. All cells were identified under CD3^+^ and CD4^+^ gates. The results are representative of five independent experiments. (**b**) The kinetics of epitope-specific T-cell responses, Th1 *(middle)* and Th17 *(right)*, and non-responsive cells *(left)* were assessed. (**c**) UreB_317–329_ and UreB_409–421_ epitope-specific Th1 and Th17 cells expressing α4β7 homing receptors were analyzed using flow cytometry. Representative flow cytometry plots of five independent experiments for α4β7 expression on the surface of specific T lymphocytes are shown. Data are shown under CD4^+^ IFN-γ^+^ and CD4^+^ IL-17^+^ gates. (**d**) UreB_317–329_ and UreB_409–421_ epitope-specific Th1 and Th17 cells expressing L-selectin chemokine receptors were analyzed using flow cytometry, and representative flow cytometric plots of five independent experiments for L-selectin expression on the surface of specific T lymphocytes are shown. Data are shown under CD4^+^ IFN-γ^+^ and CD4^+^ IL-17^+^ gates. The data are expressed as the mean ± S.D (n = 5). *P < *0.05*, **P < *0.01*, ***P < *0.001*.

Immunodominant Th1 and Th17 responses could be induced by two novel CD4^+^ T-cell epitopes, UreB_317–329_ and UreB_409–421_. However, the characteristics of these epitope-specific Th1 and Th17 cells remain unclear. Thus, lymphocytes isolated from spleens of UreB-immunized mice were cultured *in vitro* in the presence of the UreB antigen. Two epitope-specific CD4^+^ T cells were then analyzed using ICS and FCM. As shown in Fig. 4a, there were few CD4^+^ T cells secreting IFN-γ and IL-17A simultaneously after stimulation; most cells secreted either IFN-γ or IL-17A. In addition, the kinetics of the epitope-specific T-cell responses were assessed. After primary stimulation with UreB *in vitro*, Th1 responses did not significantly change during days 5 to 11, whereas the Th17 responses were continuously reduced (Fig. 4b). To investigate whether the same epitope-specific Th1 and Th17 cells could migrate back into the gastric tissue, L-selectin and α4β7 integrin, which are specific homing and chemokine receptors on CD4^+^ T cells, were examined. Our results indicated that both L-selectin and α4β7 integrin were highly expressed in epitope-specific Th1 cells, but only low expression of α4β7 integrin was found in Th17 cells (Fig. 4c,d). These results suggest that the epitope-specific Th1 lymphocytes were different from Th17 cells and that they might play different roles in protection against *H. pylori* infection.

### Two novel immunodominant epitopes trigger CD4^+^ T-cell responses and provide protection against *H. pylori* following epitope vaccination

**Figure 5:**
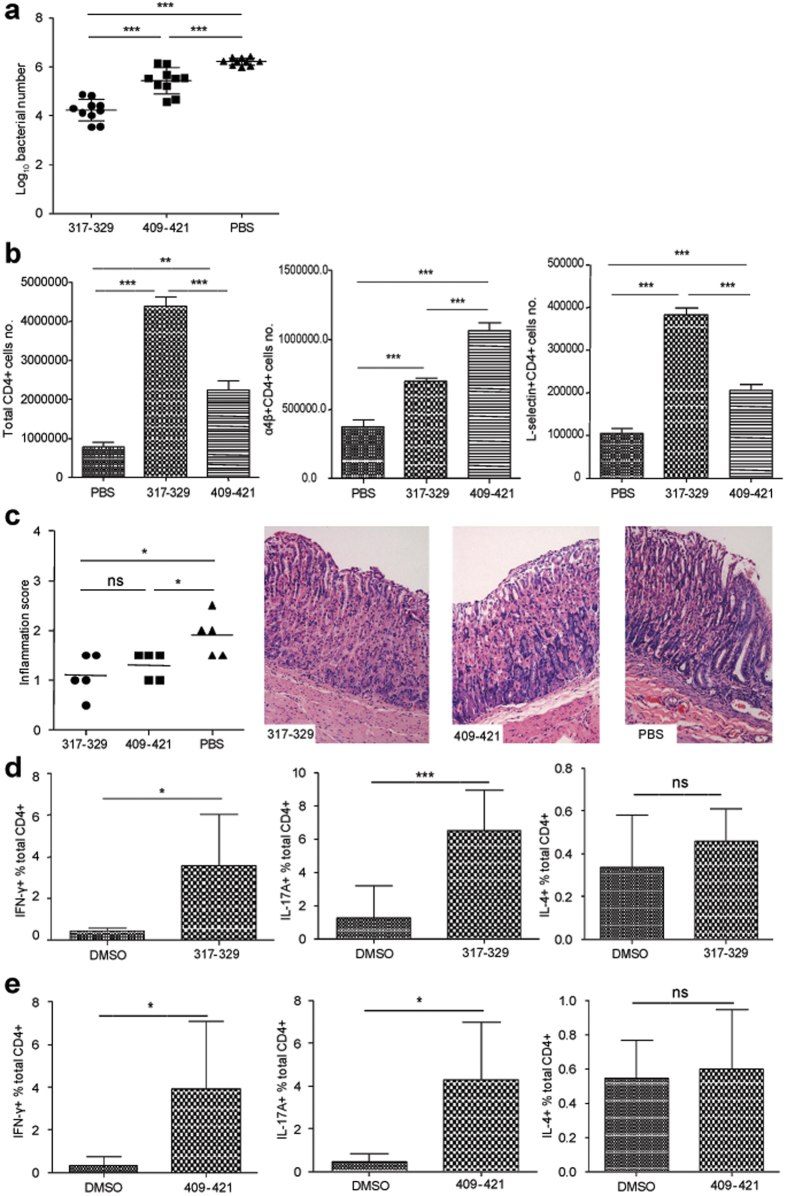
Effect of two novel immunodominant epitopes against *H. pylori* infection. (**a**) *H. pylori* colonization in the stomachs of mice immunized with peptides was determined using real-time quantitative PCR. (**b**) The number of gastric mucosal CD4^+^ T lymphocytes was analyzed using flow cytometry *(left)*. Gastric mucosal CD4^+^ T cells expressing α4β7 homing receptors were quantified *(middle)*. Gastric mucosal CD4^+^ T cells expressing L-selectin chemokine receptors were determined *(right)*. (**c**) Gastric inflammation scores of mice immunized with peptides were identified 4 weeks after infection and representative gastric histopathology of immunized mice and PBS controls are shown (H&E staining, original magnification × 200). (**d**) The epitope-specific CD4^+^ T-cell responses, including Th1, Th17 and Th2, of mice immunized with UreB_317–329_ peptide were analyzed. (**e**) The epitope-specific CD4^+^ T-cell responses, including Th1, Th17 and Th2, of mice immunized with UreB_409–421_ peptide were determined. All data were repeated more than three times. The data are expressed as the mean ± S.D (n = 10). **P < *0.01*, ***P < *0.001*.

To investigate the protective effect of two immunodominant epitopes against *H. pylori* infection, mice were immunized with either UreB_317–329_ or UreB_409–421_ and challenged with *H. pylori*. Real-time quantitative PCR was used to measure *H. pylori* colonization in the gastric mucosal four weeks post-challenge. As shown in Fig. 5a, the levels of *H. pylori* colonization in the peptide-immunized groups were considerably lower than those in PBS controls. Robust protection was observed in mice immunized with UreB_317–329_ and UreB_409–421_ (Fig. 5a), and immunization with UreB_317–329_ demonstrated a more significant protective effect than UreB_409–421_.

Next, gastric mucosal lymphocytes were isolated and analyzed using flow cytometry. These results revealed that the number of CD4^+^ T lymphocytes in the two peptide-immunized mice was significantly higher than PBS controls, and UreB_317–329_ vaccination induced greater numbers of CD4^+^ T lymphocytes than UreB_409–421_ in mucosal tissue (Fig. 5b, left). Next, the expression of L-selectin and α4β7 integrin, which plays a crucial role in lymphocytes recruitment to the gastric mucosa, was investigated. There were considerably more CD4^+^ T cells expressing these two receptors in peptide-immunized mice than in PBS controls (Fig. 5b, middle and right). Next, the pathological inflammation of gastric tissue was assessed. As shown in Fig. 5c, the inflammation of gastric tissue in peptide-immunized mice was milder than that in PBS controls after infection. There were only mild inflammatory neutrophil and mononuclear-cell infiltrates in the laminae propriae of peptide-immunized mice, whereas PBS controls showed moderate foci of neutrophil and mononuclear-cell infiltration in the lamina propria. These data suggested that the epitopes UreB_317–329_ and UreB_409–421_ may protect against the inflammation induced by *H. pylori*.

To assess whether peptide-specific T-cell responses were effectively activated, splenic lymphocytes of peptide-immunized mice were cultured *in vitro*. Seven days later, specific CD4^+^ T-cell responses were detected with UreB_317–329_ and UreB_409–421_. As shown in Fig. 5d,e, UreB_317–329_ and UreB_409–421_ stimulated CD4^+^ T cells to strongly secrete IFN-γ and IL-17A, but no IL-4 responses were observed. These results indicated that vaccination with two immunodominant epitopes provided immune protection against *H. pylori* infection.

### The same epitope-specific CD4+ T-cells secreting IFN-γ, but not CD4+ T-cells secreting IL-17A, mediate protection against *H. pylori* challenge

**Figure 6:**
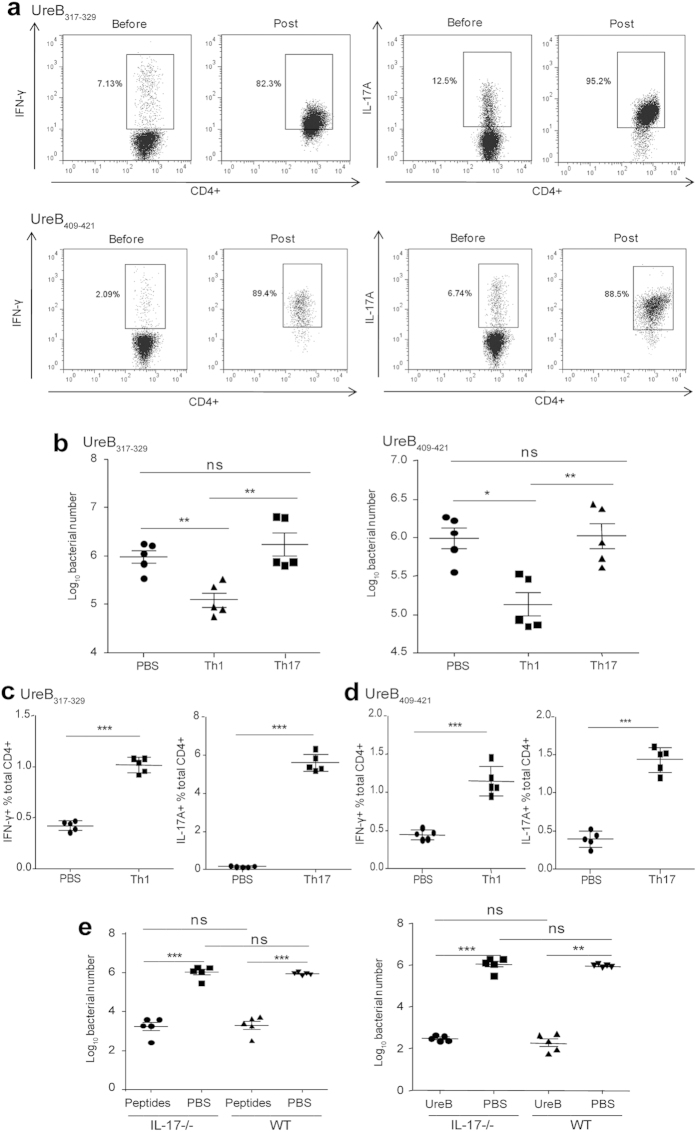
Determination of the protective effects of epitope-specific Th1 and Th17 cells. (**a**) Splenic lymphocytes of mice immunized with the peptides, UreB_317–329_ and UreB_409–421_, were cultured *in vitro* in the presence of the peptides. Then, epitope-specific Th1 and Th17 cells were isolated using a cell enrichment and detection kit, and their purities were assessed via flow cytometry. The results are representative of five independent experiments (**b**) After adoptive transfer with epitopes-specific Th1 or Th17 cells, *H. pylori* colonization of the stomachs of mice was determined. (**c**) UreB_317–329_-specific Th1 responses of mice transferred with specific CD4^+^ T cells secreting IFN-γ were analyzed *(left)*. UreB_317–329_-specific Th17 responses of mice transferred with specific CD4^+^ T cells secreting IL-17A were analyzed *(right)*. (**d**) UreB_409–421_ specific Th1 and Th17 responses of mice transferred with specific CD4^+^ T cells secreting IFN-γ and IL-17A were analyzed as described in *c*. (**e**) IL-17^−/−^ mice were immunized with peptides (UreB_317–329_ combined with UreB_407–419_) or UreB. Four weeks after *H. pylori* challenge, the bacterial colonization in the stomachs of mice immunized with peptides (left) and UreB (right) was assessed. The results of (**b**–**e**) were repeated three times. The data are expressed as the mean ± S.D (n = 5). *P < *0.05*, **P < *0.01*, ***P < *0.001*.

To further determine whether epitope-specific Th1 or Th17 cells were beneficial for protective immunity against *H. pylori* infection, splenic lymphocytes of UreB_317–329_/UreB_409–421_-immunized mice were bulk cultured *in vitro* in the presence of the peptides. UreB_317–329_ and UreB_409–421_-specific Th1 and Th17 cells were then harvested and transferred into naïve mice. As shown in Fig. 6a, the purity of UreB_317–329_-specific Th1 cells was 82.3%, and the purity of UreB_317–329_-specific Th17 cells was 95.2% after isolation. The purity of UreB_409–421_-specific Th1 cells was 89.4%, and the purity of UreB_409–421_-specific Th17 cells was 88.5%. No Tregs or other cells were detected after isolation (data not shown). *H. pylo*ri infection in the stomachs of transferred mice was analyzed four weeks after *H. pylor*i challenge. *H. pylo*ri colonization was significantly reduced in UreB_317–329_-specific Th1 cell-transferred mice, whereas no differences were detected in Th17 cell-transferred mice compared with PBS controls (Fig. 6b, left). Similar results were obtained in UreB_409–421_-specific Th1- and Th17-cell-transferred mice (Fig. 6b, right). In addition, splenic lymphocytes were isolated and analyzed to investigate whether the epitope-specific T-cell responses were present following adoptive immunity. In both UreB_317–329_ and UreB_409–421_-specific Th cell-transfer experiments, the frequencies of specific CD4^+^ T cells secreting IFN-γ or IL-17A were significant higher in transferred mice compared to PBS controls (Fig. 6c,d). These results indicated that epitope-specific Th1 immune responses mediated protection against *H. pylori* infection, whereas Th17 responses did not.

To further confirm this conclusion, IL-17^−/−^ mice were immunized with rUreB protein or peptides (UreB_317–329_ combined with UreB_407–419_) and subsequently challenged with *H. pylori* strain. As shown in Fig. 6e (left), *H. pylori* colonization was significantly reduced both in peptide-immunized IL-17^−/−^ mice and wild-type (WT) mice compared with PBS controls, whereas no differences were detected between immunized IL-17^−/−^ and WT mice. Similar results were obtained in UreB-immunized mice (Fig. 6e, right). Taken together, these results indicate that Th1 cells are sufficient to protect against the infection of *H. pylori* in the absence of IL-17A.

## Discussion

*H. pylori* colonizes the human stomach and causes a series of diseases associated with persistent inflammation^12^. However, current antibiotic therapy remains unsatisfactory^13^. Vaccination has been considered an effective method against *H. pylori* infection^14^. However, the protective effects of present vaccines are typically partial or limited. Thus, it is necessary to identify the immune responses and confirm the protective responses against *H. pylori* infection. Our studies found that the immune responses induced by UreB_317–329_ and UreB_409–421_, two novel epitopes identified in this study, provided protection against *H. pylori* challenge due to the colonization of *H. pylori* strains in the gastric mucosa, which was significantly reduced following epitope vaccination compared to controls (Fig. 5a).

Some vaccines against *H. pylori* based on B cell epitopes of urease and several B cell epitopes of urease were identified^15^. However, the function of B cells in the protective immunity remains unclear. In this study, ELISA was used to evaluate humoral immune responses in peptide-immunized mice, in whose stomachs bacteria were significantly reduced. The serum and mucosal specific antibodies levels did not differ between immunized mice and PBS controls, indicating that antibody responses were not important in *H. pylori* clearance (data not shown). However, T lymphocytes, particularly antigen-specific CD4^+^ T-cells, play a pivotal role in protection against *H. pylori* infection^16^,^17^,^18^. The transfer of cells from immunized mice to immunodeficient mice confirmed that CD4^+^ T-cells had a protective effect, whereas CD8^+^ T-cells did not^19^. In our study, we also confirmed that CD4^+^ T-cells proliferated more significantly than CD8^+^ T-cells in immunized mice, which indicated that CD4^+^ T-cell responses contributed to immune protection against *H. pylori* infection (Fig. 1).

Th1 and Th17 immune responses have been the focus of many studies in *H. pylori* infection^20^. Th1 cells have been confirmed to contribute to the elimination of *H. pylori*^16^,^17^, but the role of the Th17 cells remains controversial. The effects of vaccination-induced Th17 cells were confirmed to be preventive against some microbes, including *Klebsiella pneumonia*^21^, *Streptococcus pneumoniae*^22^, and *Blastomyces dermatitidis*^23^. Th17 cells could recruit neutrophils and macrophages, initiate humoral immunity, and augment Th1 cells to mediate host defense against microorganisms^24^. Thus, it is reasonable to hypothesize that Th17 cells play a role in the vaccine-induced *H. pylori* clearance^25^. However, the results of this study indicated that Th17 cells were not required for the protective effects of immunization against *H. pylori* (Fig. 6). The data of IL-17^−/−^ mice further confirmed this finding (Fig. 6e). During *H. pylori* infection, significant IL-17 responses were detected in mouse models and human patients^26^,^27^,^28^. The increased IL-17 attracts neutrophils to the stomach, which kill *H. pylori* in the early phase^29^,^30^. IL-17 neutralization causes an increase in *H. pylori* colonization^31^. These findings suggest that IL-17 plays a protective role against *H. pylori*. However, IL-17 cannot clear *H. pylori* completely, and the persistence of IL-17 ultimately results in gastritis, ulcer and some other diseases^32^,^33^,^34^. Therefore, IL-17 may play a dual role in infection and vaccination. In infection, the Th17 cell responses were suppressed by Treg cells, thereby leading to bacterial persistence and gastritis. Immunization enhances Th17 cell responses, which can overcome Treg-cell suppression and affect bacterial clearance^35^. Although this perspective is useful, data obtained from IL-17^−/−^ mice indicated that vaccine-induced immunity against *H. pylori* can function in the absence of IL-17A^36^,^37^,^38^. Thus, the role of Th17 cells in *H. pylori*, infection, which causes chronic diseases, remains uncertain. In this study, we demonstrated that Th1 immune responses mediated protection against *H. pylori* infection, whereas Th17 responses did not. In the case of UreB- or peptide-immunized mice, we hypothesize that Th1 cells are sufficient to protect against *H. pylori* infection. Additional studies are needed to confirm the effect of Th17 cells during the course of *H. pylori* infection and the interaction between Th17 and Th1 cells in particular.

In this study, Th1 and Th17 immune responses were focused in *H. pylori* infection (Fig. 1) and immunodominant Th epitopes of UreB were screened and confirmed (Fig. 2). Although three H-2^d^-restricted Th epitopes from UreB predicted using software were identified in our previous study^11^, the results predicted by software are often inaccurate and incomplete. We confirmed the presence of an immunodominant epitope that differed from our previous results. Thus, we used a systematic method to map epitopes from the *H. pylori* antigen UreB and two novel Th epitopes, UreB_317–329_ and UreB_409–421_, which induced stronger responses than the three previously identified peptides (Fig. 3b,e). In addition, the epitopes of UreB, UreB_317–329_ and UreB_409–421_, were not accurately predicted via software (data not shown). Importantly, UreB_317–329_ and UreB_409–421_ eliminated the bacterial colonization and protected against the inflammation induced by *H. pylori*. Furthermore, the two epitopes induced Th1 and Th17 responses simultaneously following peptide vaccination. To examine the contribution of Th1 and Th17 responses to the observed protective effects, epitope-specific Th1 and Th17 cell were isolated, and their purities were confirmed to be satisfactory (Fig. 6a). Then, adoptive transfers were performed. The specific T cells were confirmed to be stable post-transfer (Data were not shown). Finally, we determined that epitope-specific Th1 cells had a protective effect against *H. pylori* infection, whereas Th17 cells did not (Fig. 6).

The homing and chemokine receptors, L-selectin and α4β7 integrin, respectively, expressed on the T cell surface are involve in the migration of lymphocytes from peripheral tissue to inflammatory sites^39^. Specific T lymphocytes home to gastric mucosa and play a protective effect against *H. pylori* via homing receptors^40^,^41^. In this study, we confirmed that a larger number of antigen-specific T cells secreting IFN-γ expressed homing and chemokine receptors compared to T cells secreting IL-17A (Fig. 4c,d), and the Th1 immune responses demonstrated a protective effect (Fig. 6). Abundant gastric mucosal CD4^+^ T lymphocytes expressing homing and chemokine receptors were detected following epitope vaccination (Fig. 5b). These findings indicated that immunodominant epitopes induced specific Th1, but not Th17 lymphocytes, to recruit into the gastric mucosa to exert effects on the elimination of *H. pylori* colonization.

In conclusion, we have demonstrated that antigen-specific Th1 and Th17 immune responses were induced in immunized mice. Furthermore, using a systematic approach, two novel immunodominant Th epitopes of UreB antigen, UreB_317–329_ and UreB_409–421_, were identified, and their profiles were characterized. The two peptides induced both Th1 and Th17 responses. The colonization of *H. pylori* and the inflammation in the gastric mucosa were significantly reduced following epitope vaccination. However, only epitope-specific Th1 responses were confirmed to play a protective role in *H. pylori* infection. In addition, homing and chemokine receptors may contribute to the protective immunity of CD4^+^ T-cells secreting IFN-γ. The data obtained in this study will help to further understand and characterize the immune response to *H. pylori* infection. However, *H. pylori* exhibits a large genome that encodes many antigens. Thus, further research is needed to identify the most immunodominant antigens and appreciate its interaction with the human host avoiding bacterial immune escape.

## Methods

### Recombinant antigen, synthetic peptides, and antibodies

Recombinant urease subunit B (rUreB) protein was expressed in *Escherichia coli* as previously described^11^ and stored at −80 °C. The following 93 18-mer peptides overlapping by 12 aa and 13-mer peptides overlapping by 11 aa were synthesized by GL Biochem (Shanghai, China). The purity (>90%) was assessed using high-pressure liquid chromatography, and the molecular weights of the peptides were determined via mass spectrum analysis. All peptides were dissolved in DMSO (Sigma) and stored in aliquots at −80 °C. Anti-mouse CD3 (FITC), anti-mouse CD4 (APC), anti-mouse α4β7 (FITC), anti-mouse L-selectin (PE-Cy7), anti-mouse IFN-γ (PE), anti-mouse IFN-γ (PerCP-Cy5.5), anti-mouse IL-17A (PE) and anti-mouse IL-17A (PerCP-Cy5.5) were purchased from Biolegend. Anti-mouse MHC class I (H-2Kd/H-2Dd), anti-mouse MHC class II (I-A) and anti-mouse MHC class II (I-Ek) were purchased from eBioscience.

### *H. pylori* culture

The BALB/c mouse-adapted *H. pylori* strain B6^42^ was grown on brain-heart infusion plates containing 10% rabbit blood under microaerobic conditions. After 2 days, the *H. pylori* strain was amplified in Brucella broth with 5% fetal bovine serum under gentle shaking at 37 °C. The concentration of *H. pylori* strain was adjusted to 10^9^ colony-forming units (CFU)/ml prior to inoculation.

### Mice, immunization and infection

Six- to eight-week-old SPF female BALB/c mice were purchased from the Experimental Animal Center of the Third Military Medical University. IL-17^−/−^ mice (C57BL/6 background) were bred in our laboratory. Animal maintenance and experimental procedures were carried out in accordance with the National Institutes of Health Guidelines for the Use of Experimental Animals and approved by the Medicine Animal Care Committee of the Third Military Medical University.

Mice were immunized with 100 μg of rUreB protein emulsified in equivoluminal Complete Freund’s adjuvant subcutaneously in four limbs. Immunization was boosted 2 weeks later with equivalent protein combined with incomplete Freund’s adjuvant. After 2 weeks, rUreB protein without adjuvant was used for the last vaccination. For peptide immunization, the mice were immunized with 50 μg of peptides in 20 μg of CpG OND 1826 (Invivogen), a mucosal adjuvant, via an intranasal route four times over one-week intervals. As controls, the mice were immunized with PBS combined with adjuvants instead of protein or peptides using the same procedure.

One week after the final boost, the mice were infected with 2 × 10^8^ CFU of *H. pylori* strain using intubation for four times over a one-day interval. The mice were euthanized four weeks after the last intragastric administration. The immune responses and *H. pylori* colonization were assessed.

### Determination of inflammation and *H. pylori* colonization

Four weeks after the last infection, the mice were euthanized. The stomachs were cut along the greater curvature. Half of the stomach was collected, fixed with 4% paraformaldehyde for 24 h and then embedded in paraffin. Each specimen of all of the tested mice was sectioned for three slides and stained with hematoxylin and eosin. The inflammation of all slides was evaluated and graded independently by two pathologists according to the criteria. The inflammation scores depend on the infiltrate of inflammatory cells, epithelial hyperplasia, and mucous cell metaplasia, among other factors, similar to the criteria in previous study^43^. The remaining stomach was used to determine colonization of *H. pylori* in the stomach using real-time quantitative PCR. An analysis of *H. pylori* 16S rDNA was performed according to previously described methods^38^.

### Preparation of antigen presenting cells (APCs) and the CD4^+^/CD8^+^ T lymphocyte proliferation assay

CD19^+^ cells obtained from WT mice were harvested from spleens using a mouse CD19^+^ isolation kit (Miltenyi Biotec) and then used as APCs for subsequent experiments. The purity of the isolated cells was >95%, as determined using flow cytometry. APCs were resuspended in RP-10 consisting of RPMI-1640 medium (GIBCO) supplemented with 10% FCS, 2-ME (5 × 10^−5^ M) and antibiotics (100 U/ml penicillin, 100 μg/ml streptomycin).

Spleens and stomach-draining lymph nodes were isolated from mice immunized with UreB and dissociated into a single-cell suspension. Then, CD4^+^/CD8^+^ T-cells were sorted using the mouse CD4^+^/CD8^+^ isolation kit (Miltenyi Biotec) according to the manufacturer’s instructions. The purity of the isolated cells was routinely found to be >95%. The isolated APCs (2 × 10^5^ cells/well) were then pulsed with UreB antigen (0.5 μM) in 96-well flat-bottom plates in a CO_2_ incubator at 37 °C. Cultures with OVA protein were used as negative controls. One day later, the cells were irradiated by Co^60^ (20 Gy) and cocultured with isolated CD4^+^/CD8^+^ T-cells (4 × 10^5^ cells/well) in 96-well U-bottom culture plates for 4 days in triplicate. During the final 16–18 h, 1 μCi [^3^H] thymidine (^3^H-TdR) was added to each well. Radioactivity, which reflected cell proliferation, was measured using a liquid scintillation counter. The results are expressed as the stimulated indices (SIs), which were defined as the mean cpm ratio of stimulated cultures to negative control cultures.

### Splenic T lymphocyte isolation and RT-PCR

CD4^+^ and CD8^+^ T lymphocytes were isolated from the spleens of immunized mice according to the protocol provided by the Miltenyi Biotec Company. RNA was extracted from splenic CD4^+^ or CD8^+^ T lymphocytes using the Tripure RNA isolation kit (Roche) according to the manufacturer’s instructions. The RNA was then reverse transcribed, and the cDNA was amplified using specific primers for IL-4 (sense primer: 5′-GAGCTGCAGAGACTCTTTCG-3′, antisense primer: 5′-ACTCATTCATGGTGCAGCTTA-3′), IFN-γ (sense primer: 5′-GATCCTTTGGACCCTCTGACTT-3′, antisense primer: 5′-TGACTGTGCCGTGGCAGTAA-3′), IL-17A (sense primer: 5′-CTCCAGAAGGCCCTCAGACTAC-3′, antisense primer: 5′-GGGTC TTCATTGCGGTGG-3′) and β-actin (sense primer: 5′-CCTGCAGAGTTAAGCATGCCAG-3′, antisense primer: 5′-TGCTTGATCACATGTCTCGATCC-3′). The assay was performed using the Bio-Rad iQ5 multicolor Real-time PCR Detection System following relative quantification.

### Specific T cell bulk culture

Spleens of immunized mice were harvested, and the lymphocytes were isolated using a Ficoll-Hypaque (TBDscience, Tianjin, China) gradient. Next, isolated lymphocytes were pulsed with rUreB protein (0.5 μM)/peptides (5 μM) and stimulated with 5 U/ml rmIL-2 (PeproTech, Rocky Hill, NJ, USA) in RP-10. Dead cells were removed using a Ficoll-Hypaque gradient on day 5, and the lymphocytes were collected and cultured in RP-10 containing 20 U/ml rmIL-2. Half of the medium was removed when it turned yellow and then replaced with fresh RP-10 containing 20 U/ml rmIL-2. Lymphocytes were harvested and analyzed at specific times.

### Generation of bone marrow-derived dendritic cells (DCs) and co-culture with immunodominant epitope-specific T cells

Bone marrow was harvested from the femurs of WT BALB/c mice. After treatment with erythrocyte lysis buffer, the cells were cultured in RP-10 at a concentration of 10^6^/ml in the absence of rmIL-2. Two hours later, the nonadherent cells were removed, and fresh RP-10 supplemented with 200 U/ml recombinant murine GM-CSF and 5 U/ml recombinant murine IL-4 was added. Eight days later, the nonadherent cells (DCs) were harvested and pulsed with 0.5 μM rUreB antigen for 1 h in fresh RP-10 and washed. Then, the DCs were co-cultured with epitope-specific T-cells at a ratio of 1:5 for 5 h in the presence of brefeldin A.

### Intracellular cytokine staining (ICS)

In the 18-mer and 13-mer screening assays, cultured lymphocytes were harvested and incubated with peptides at 5 μM in RP-10 for 5 h in the presence of brefeldin A. In the MHC-restricted determination assays, lymphocytes were initially incubated with specific MHC antibodies for 30 min, washed and then pulsed with peptides for 5 h as previously described. Next, the cells were collected and labeled with anti-CD3-FITC (or anti-α4β7-FITC), anti-CD4-APC and anti-L-selectin-PE-Cy7 at 4 °C for 30 min, washed and fixed with 4% paraformaldehyde at 4 °C for 20 min. Finally, the cells were washed and labeled with anti-IFN-γ-PE and anti-IL-17A-PerCP-Cy5.5 in 0.2% saponin. Approximately 100,000 cells were acquired using the FACSCanto II flow cytometer (Becton Dickinson), and the FCS files were analyzed using FlowJo software.

### Harvesting of gastric mucosal lymphocytes

Mice were euthanized 4 weeks post-infection. The stomachs were immediately removed and rinsed with normal saline. The stomachs were then treated with Hanks Balanced Salt Solution without Ca^2+^ and Mg^2+^ and supplemented with 1 mM EDTA, 1 mM DTT and 2% FCS for 50 min at 37 °C. Supernatant containing the cells isolated from the gastric mucosa was collected and centrifuged. The cells were resuspended in PBS after filtration and labeled with anti-CD4-APC, anti-α4β7-FITC and anti-L-selectin-PE-Cy7. The cells were then acquired and quantified using the FACSCanto II flow cytometer (Becton Dickinson). The FCS files were analyzed using FlowJo software.

### Adoptive transfer and challenge experiments

Epitope-specific Th1 (or Th17) lymphocytes were isolated using the Mouse IFN-γ Secretion Assay Cell Enrichment and Detection Kit (or Mouse IL-17A Secretion Assay Cell Enrichment and Detection Kit, Miltenyi Biotec) according to the manufacturer’s instructions. Briefly, splenic lymphocytes of UreB_317–329_/UreB_407–419_ immunized mice were bulk cultured *in vitro* in the presence of the peptides. Cells were then harvested and CD4^+^ T cells were enriched using commercial MACS beads designed to negatively select CD4^+^ T cells (Miltenyi Biotec). The isolated CD4^+^ T cells were stimulated with peptides for 5 h. Cells were then labeled with Mouse IFN-γ Catch Reagent (or IL-17A Catch Reagent), which could attach to the cell surface. Subsequently, the cells were incubated briefly at 37 °C to allow for cytokine secretion. The secreted IFN-γ (or IL-17A) bound to the IFN-γ Catch Reagent (or IL-17A Catch Reagent) on positive, secreting cells. These cells were then labeled with a second IFN-γ specific antibody (or IL-17A specific antibody) conjugated to phycoerythrin (PE). Finally, the IFN-γ- (or IL-17A)-secreting cells were magnetically labeled with anti-PE MicroBeads and enriched over a MACS Column. Specific lymphocytes were collected, centrifuged, resuspended in normal saline, and injected i.v. into mice (10^6^ cells/mouse in 200 μl of normal saline). The mice were challenged with *H. pylori* on the following day.

### Statistical analysis

Results of *H. pylori* colonization in the stomach, proliferation assays and levels of cytokines were analyzed using the unpaired Student *t*-test. Other data were analyzed using one-way ANOVA followed by Newman-Keuls test for dependent variables. All data were analyzed using the SPSS (Statistical Package for the Social Sciences) 13.0 statistical program and are expressed as the mean ± standard deviation (S.D). Values of P ≤ 0.05 were considered statistically significant.

## Additional Information

**How to cite this article**: Li, B. *et al*. Immunodominant Epitope-Specific Th1 But Not Th17 Responses Mediate Protection against *Helicobacter pylori* Infection following UreB Vaccination of BALB/c mice. *Sci. Rep*. **5**, 14793; doi: 10.1038/srep14793 (2015).
